# Spatial associations of long-term exposure to diesel particulate matter with seasonal and annual mortality due to COVID-19 in the contiguous United States

**DOI:** 10.1186/s12889-023-15064-5

**Published:** 2023-03-03

**Authors:** Martine Elisabeth Mathieu, Joshua Gray, Jennifer Richmond-Bryant

**Affiliations:** 1 Center for Geospatial Analytics, Department of Forestry and Environmental Resources, North Carolina State University, 2800 Faucette Drive, Raleigh, NC 27695-8008 USA; 2 Department of Forestry and Environmental Resources, North Carolina State University, Raleigh, NC 27695-8008 USA

**Keywords:** Diesel Particulate Matter, COVID-19 mortality, Global models, Geographically weighted regression, Spatial pattern, Seasonal effects

## Abstract

**Background:**

People with certain underlying respiratory and cardiovascular conditions might be at an increased risk for severe illness from COVID-19. Diesel Particulate Matter (DPM) exposure may affect the pulmonary and cardiovascular systems. The study aims to assess if DPM was spatially associated with COVID-19 mortality rates across three waves of the disease and throughout 2020.

**Methods:**

We tested an ordinary least squares (OLS) model, then two global models, a spatial lag model (SLM) and a spatial error model (SEM) designed to explore spatial dependence, and a geographically weighted regression (GWR) model designed to explore local associations between COVID-19 mortality rates and DPM exposure, using data from the 2018 AirToxScreen database.

**Results:**

The GWR model found that associations between COVID-19 mortality rate and DPM concentrations may increase up to 77 deaths per 100,000 people in some US counties for every interquartile range (0.21 μg/m^3^) increase in DPM concentration. Significant positive associations between mortality rate and DPM were observed in New York, New Jersey, eastern Pennsylvania, and western Connecticut for the wave from January to May, and in southern Florida and southern Texas for June to September. The period from October to December exhibited a negative association in most parts of the US, which seems to have influenced the year-long relationship due to the large number of deaths during that wave of the disease.

**Conclusions:**

Our models provided a picture in which long-term DPM exposure may have influenced COVID-19 mortality during the early stages of the disease. That influence appears to have waned over time as transmission patterns evolved.

**Supplementary Information:**

The online version contains supplementary material available at 10.1186/s12889-023-15064-5.

## Background

In 2020, more than 20 million cases of coronavirus disease 2019 (COVID-19) were identified in the United States (U.S.), and more than 350,000 people died [5, 9, 19]. In addition to age, socioeconomic status, access to healthcare, physical environment, and education have been identified as social determinants of COVID-19 hospitalization and mortality [30, 36]. Several studies have observed a disproportionate share of COVID-19 incidence and mortality among predominantly Black U.S. communities, which may be partly attributable to social and economic inequalities and preexisting comorbidities [7, 8, 33 36, 40, 55], as well as to disproportionately high exposures to air pollution [29].

The impact of particulate matter exposures on COVID-19 outcomes have also been evaluated, with some studies centered on diesel particulate matter (DPM). In an investigation of the role of long-term exposure (2000-2016) to air pollution during the first months of the pandemic, Wu et al. [54] found that an increase of 1 μg/m^3^ in particulate matter with a nominal diameter of 2.5 μm (PM_2.5_) was associated with an 11% increase in the COVID-19 death rate for January 1-June 18, 2020. Bozack et al. [3] performed a similar analysis to test associations of COVID-19 intensive care unit (ICU) admission and mortality with long-term concentrations of PM_2.5_, nitrogen dioxide, and black carbon for the period March 8-August 30, 2020 in New York City. They noted an association of ICU admission and mortality with long-term PM_2.5_ concentrations (collected December, 2018-December, 2019). Petroni et al. [35] investigated the association of COVID-19 mortality with respiratory hazard index calculated across 3223 U.S. counties using emissions data for 2014 and COVID-19 data through May 13, 2020. They observed a 9% increase in COVID-19 mortality per unit increase in respiratory hazard index, which includes DPM. Their analyses with only DPM demonstrated an increased effect of 182% in the mortality rate ratio with a 0.5 μg/m^3^ increase in DPM concentration. Hendryx and Luo [18] studied the association of long-term exposure to ozone (obtained from 2016), PM_2.5_ (obtained from 2016), and DPM (obtained from 2014) with COVID-19 prevalence and mortality through May 31, 2020. They showed an increase of 14.3 deaths per 100,000 U.S. residents for each DPM concentration increase of 1 μg/m^3^ in a single-pollutant model adjusted for demographic, health, smoking, and COVID-19 testing covariates. These findings collectively suggest that long-term PM exposure may predispose an individual to COVID-19 mortality. However, association of COVID-19 mortality with long-term DPM may change over time with the evolution of the coronavirus and changes in policies and personal behaviors. Our understanding of the effect of long-term DPM exposure on COVID-19 mortality during different waves of the disease and over the locations impacted by those waves remain unknown, hampering anticipation of disease hotspots.

DPM is composed of a complex mixture of black carbon and organic carbon. Studies have shown that 80-90% of particles emitted by diesel engines are smaller than 2.0 μm [12, 24], small enough to penetrate the alveoli [41]. Long-term DPM exposure has been associated with adverse respiratory and cardiovascular effects [12, 37, 41]. Diesel engines power school buses, heavy-duty trucks, a variety of off-road heavy equipment, shipping, and commercial boating [12, 24]. DPM emissions are higher in urban areas, where most of the global population lives [12, 41]. Likewise, greater DPM concentrations have been observed in socioeconomically disadvantaged communities [8, 12].

## Methods

Our study explores spatial associations between long term average concentrations of DPM, as a metric for past air pollution exposure, and COVID-19 mortality across each pandemic wave and throughout 2020 in the U.S. The objectives of the study are 1) to assess if living near DPM sources increased the risk of death from COVID-19, 2) to estimate how associations between mortality and long-term exposure to DPM (using the U.S. Environmental Protection Agency [50] broad definition of long-term exposure measured over “months to years”) may have changed over time with changes in the Coronavirus and in the population’s behavior, and 3) to test if models accounting for spatial autocorrelation improve model estimates. Data for air pollution, health, demographic, and social determinants of health were merged for this analysis, and global and local models were both applied to examine these relationships.

### Population data

Two measures of mortality were considered for our study: mortality rate (defined as the number of dead per 100,000 people in a defined geographic area) and case fatality rate (CFR, defined as the percent dead among confirmed COVID-19 cases in a defined geographic area). County-level number of COVID-19 deaths and CFR were obtained from the publicly-available Johns Hopkins Coronavirus Resource Center [19] for the period January 1- December 31, 2020. Total population shapefile data were obtained from the U.S. Census Bureau [49] for the mortality rate calculation. Use of the mortality rate in our model may point to an impact of long-term DPM exposure on COVID-19 mortality in the general population. Use of the CFR in our models may indicate an effect of long-term DPM exposure on mortality among those who are already infected with COVID-19. An advantage of the CFR is that positive associations may indicate that long-term DPM exposure causes death due to COVID-19. However, a disadvantage of the CFR is that two measures (COVID-19 cases and deaths) are estimated, so it is susceptible to errors due to undercounting both the COVID-19 mortality count and the COVID-19 case count. The mortality rate is only susceptible to errors in the death count.

Data for potential confounders associated with the measures of COVID-19 mortality and DPM, including access to health care, education, poverty, demographics, transportation, and occupation were obtained from the American Community Survey (ACS [48];) and the County Health Rankings (CHR [42];) (Table 1). The variables tested as potential confounders are similar to those used in other studies investigating factors associated with COVID-19 that also tested for confounders and observed associations with variables relating to socioeconomic status, demographics, and healthcare availability that could potentially be correlated with air pollution (e.g., [30, 44]).

**Table 1:** Potential confounders tested in the models

**Race/ethnicity**	**Education**
Fraction Black	Fraction Incomplete school
Fraction White	**Poverty and Wealth**
Fraction Hispanic	Fraction unhoused
Fraction American Indian	Fraction with a severe housing burden (more than 50% of monthly income spent on housing)
Fraction Asian	Fraction with one of four housing problems: overcrowding, high costs, lack of kitchen, lack of plumbing
Fraction Pacific Islander	Food-environment index
**Transportation**	Income inequality (ratio of 80th percentile to 20th percentile)
Fraction who walks to work	Fraction unemployment
Fraction who takes public transportation to work	Fraction median income
Fraction who takes a bicycle or motorcycle to work	Fraction in poverty
Fraction who drives a car to work	Median home value
Fraction average time to work	**Demographics**
Traffic volume	Population density
**Age**	
Fraction over 65	
Median age	
**Occupation**	
Fraction in a service occupation	
Fraction in a manual occupation	
Fraction working in a mining or agricultural occupation	
Fraction working in a construction occupation	
Fraction working in a utilities occupation	
**Health**	
Fraction in poor health	
Fraction obese	
Fraction with diabetes	
Fraction reporting inactivity (no leisure time physical activity in the past month)	
Fraction smoking	
**Access to Healthcare**	
Fraction uninsured	
Fraction population receiving health care	
Fraction hospitals per county	
Fraction hospital beds available	

### Exposure data

Long-term average DPM concentrations were obtained from the 2018 AirToxScreen database, the most recently modeled concentrations of hazardous air pollutants and select other pollutants [51]. EPA used a hybrid model that coupled a Community Multiscale Air Quality (CMAQ) chemical transport model to the American Meteorological Society/Environmental Protection Agency Regulatory Model (AERMOD), a dispersion model, to estimate AirToxScreen air pollutant concentrations at the census tract level through a multi-step process. CMAQ v5.2 was first run over a 12 km × 12 km grid based on DPM emissions inputs from the National Emissions Inventory [51]. Next, the AERMOD dispersion model was run for each source using the same inputs but with receptors distributed over census tract centroids. Finally, concentrations estimated by AERMOD along the census tract centroids were scaled by the ratio of the CMAQ concentration to the average of the AERMOD concentrations over that same grid cell. This formulation allows for more accurate representation of the chemistry and physics of the DPM than the AERMOD dispersion model can provide alone, while maintaining the finer census tract level spatial resolution of the dispersion model. Because the concentrations are calculated from the annual emissions, the concentrations are annual averages.

### Model runs

We tested the associations of COVID-19 mortality rate and CFR with long-term DPM concentrations across the contiguous United States for time periods coinciding with each COVID-19 wave in 2020: January 1-May 31, 2020, June 1-September 30, 2020, and October 1-December 31, 2020. We also ran the models for the entire year: January 1-December 31, 2020.

We used regression analysis to examine spatial non-stationarity in the relationship between the measures of COVID-19 mortality and DPM while accounting for potentially confounding effects. This work is similar to spatial modeling approaches used by Sun et al. [47] and Rahman et al. [39]. Sun et al. [47] investigated different spatial regression models and compared them with an ordinary least squares (OLS) regression model to explain the transmission pattern of COVID-19. County-level race/ethnicity and socio-economic covariates were included in their models. We adapted their approach by focusing on associations of COVID-19 mortality with DPM and by investigating different waves of the disease. Three global models, OLS, spatial lag model (SLM), and spatial error model (SEM), were run to produce a nationwide effect estimate. One local model, geographically weighted regression (GWR), produced effect estimates at the county scale. The R Statistical Software version 4.0.5 was used to run all code. We performed spatial regression modeling with the following libraries: *spdep*, *spgwr*, and *spatialreg*.

OLS models are designed to minimize the sum of squared differences between the true data and the prediction across the dataset [17]. Mollalo et al. [30] studied county-level variations of COVID-19 incidence in the U.S. From a list of 35 demographic, socio-economic, topographic and environmental variables, they used a stepwise forward selection procedure and then checked for multicollinearity to determine the most significant predictors of COVID-19. Then, using the same selected explanatory variables, they tested their model using OLS and several spatial models including SEM, SLM, and GWR (described below). Accounting for spatial autocorrelation in their model improved performance over OLS. Karaye and Horney [20] also compared OLS to spatial regression models to analyze the impact of social vulnerability on COVID-19 cases. Spatial autocorrelation of the residuals may compromise the validity of the OLS model and produce biased estimators [25, 28]. The model assumptions of zero mean, independence, heteroscedasticity, and normal distribution are met for the case where OLS is a complete and correct model in which the variables capture all of the spatial variation without specifying spatial positions [10, 43]. Spatial autocorrelation in residuals may occur due to an omitted variable. Heteroscedasticity, or dependence of the residuals on the fitted values, may result in part from spatial autocorrelation [28]. This was evaluated in the OLS using the Breusch-Pagan test for heteroscedasticity of the residuals. The SLM and SEM employ generalized frameworks that apply a transformation to the data to improve heteroscedasticity of the data using appropriate control of the error term and calculate efficient maximum-likelihood estimates [4].

SLMs estimate an autocorrelation parameter (“spatial lag”) using a weighted average of the response variable across neighboring areas, testing if neighboring observations affect one another [16, 26, 47]. As the autocorrelation parameter approaches zero, the SLM approaches the OLS [30]. In SEMs, errors across neighboring areas are autocorrelated (“spatial error”) [16, 23]. SEMs estimate the relationship between the residuals in a spatial region and those in adjacent regions [47]. The spatial structure is in the residuals, meaning that some important predictors are omitted in the model [6].

SLM and SEM have only one spatial dependence parameter. The single-valued characteristic makes it impossible for global spatial models to reveal local spatial patterns [6, 14]. Another limitation of global spatial models is that the model is dependent on the spatial weighting matrix 6. In contrast, GWR allows for local models to be fit to each observation using spatial distance as a weighting factor for the influence of all other points [14]. To determine local associations between COVID-19 cases in the U.S. and demographic, socio-economic, topographic and environmental parameters, Mollalo et al. [30] examined two local models including GWR. The variables incorporated in the model are the same set used for OLS, SLM, and SEM. Similarly, Karaye and Horney [20] compared GWR to OLS to understand the spatially varying effect in the relationship between social vulnerability and COVID-19 case counts. The main advantage of GWR as a local model is the ability to test for spatial variability among the effects of different variables in the model [6, 14, 25]. Another strength is that GWR has the same model structure as the OLS, which facilitates comparison between the two models [14].

For our spatial autoregressive models, we estimated spatial relationships between regions based on contiguous boundaries shared between 2 or more counties, assuming that COVID-19 spread in a county is influenced by adjacent counties. ﻿For GWR, a cross-validation function minimizes the root mean square prediction error that defines the weight matrix. We evaluated spatial autocorrelation among contiguous cells in the model residuals using Moran’s I [31]. Statistically significant Moran’s I indicates either correlation or anticorrelation among neighboring units. Additionally, we used Lagrange multiplier test statistics to understand whether the spatial lag or spatial error pattern is more important for interpreting the local results.

The level of urgency of the COVID-19 outbreak contributed to uncertain policy decisions and interventions in health in compressed timeframes coupled with the complex social, economic and political events of 2020 [22]. Effects related to pandemic waves could have influenced the importance of specific variables during these different times of the year. Therefore, a set of different covariates have been integrated into the model for each time period. To determine which covariates to include in the regression models of COVID-19 mortality, we applied a stepwise selection algorithm for each season (Table 1). Then, the same covariates were incorporated in the best model for OLS, SLM, SEM, and GWR for each specific wave (Table 2), based on the following framework:

1 $$\textrm{COVID}-19\ \textrm{deaths}=\textrm{DPM}\ \textrm{concentration}+\textrm{Confounder}\ \textrm{variables}+\textrm{error}\ \textrm{term}$$

**Table 2:** Model framework for each wave modeled

Wave Dates	Models
Mortality Rate	
Jan 1-May 31, 2020	COVID-19 deaths ~ log(DPM concentration) + Fraction Black + Fraction American Indian + Fraction who take public transportation to work + Fraction uninsured + Fraction smoking + Fraction income inequality + Population density (2)
Jun 1-Sep 30, 2020	COVID-19 deaths ~ log(DPM concentration) + Fraction Black + Fraction Hispanic + Fraction who take public transportation to work + Fraction who drive to work + Fraction reporting inactivity + Fraction incomplete school + Population density (3)
Oct 1-Dec 31, 2020	COVID-19 deaths ~ log(DPM concentration) + Fraction Black + Fraction American Indian + Fraction working in a mining or agricultural occupation + Fraction smoking + Fraction obese + Fraction over 65 + Fraction experiencing housing problems (4)
Jan 1-Dec 31, 2020	COVID-19 deaths ~ log(DPM concentration) + Fraction Black + Fraction Hispanic + Fraction American Indian + Fraction working in a mining or agricultural occupation + Fraction reporting inactivity + Fraction income inequality + Fraction experiencing housing problems (5)
Case Fatality Rate	
Jan 1-May 31, 2020	Case Fatality Rate ~ log(DPM concentration) + Unemployment + Income inequality + Severe housing burden + Fraction working in a mining or agricultural occupation + Fraction working in construction + Fraction incomplete school + Fraction Pacific Islander
Jun 1-Sep 30, 2020	Case Fatality Rate ~ log(DPM concentration) + Unemployment + Fraction income inequality + Fraction who take public transportation to work + Fraction working in a mining or agricultural occupation + Fraction of time spent commuting to work + Fraction Hispanic + Fraction Pacific Islander
Oct 1-Dec 31, 2020	Case Fatality Ratio ~ log(DPM concentration) + Fraction uninsured + Unemployment + Fraction reporting inactivity + Fraction who take public transportation to work + Fraction working in a mining or agricultural occupation + Fraction of time spent commuting to work + Fraction Pacific Islander
Jan 1-Dec 31, 2020	Case Fatality Rate ~ log(DPM concentration) + Fraction uninsured + Fraction reporting inactivity + Unemployment + Fraction income inequality + Fraction who take public transportation to work + Fraction working in a mining or agricultural occupation + Fraction Pacific Islander

The confounder selection procedure was based on minimizing the Akaike information criterion (AIC) after controlling for multicollinearity. We used this same process for each of the three waves and throughout 2020 to find the most significant models for determining the nationwide and local associations between COVID-19 mortality and DPM concentration.

## Results

**Fig. 1:**
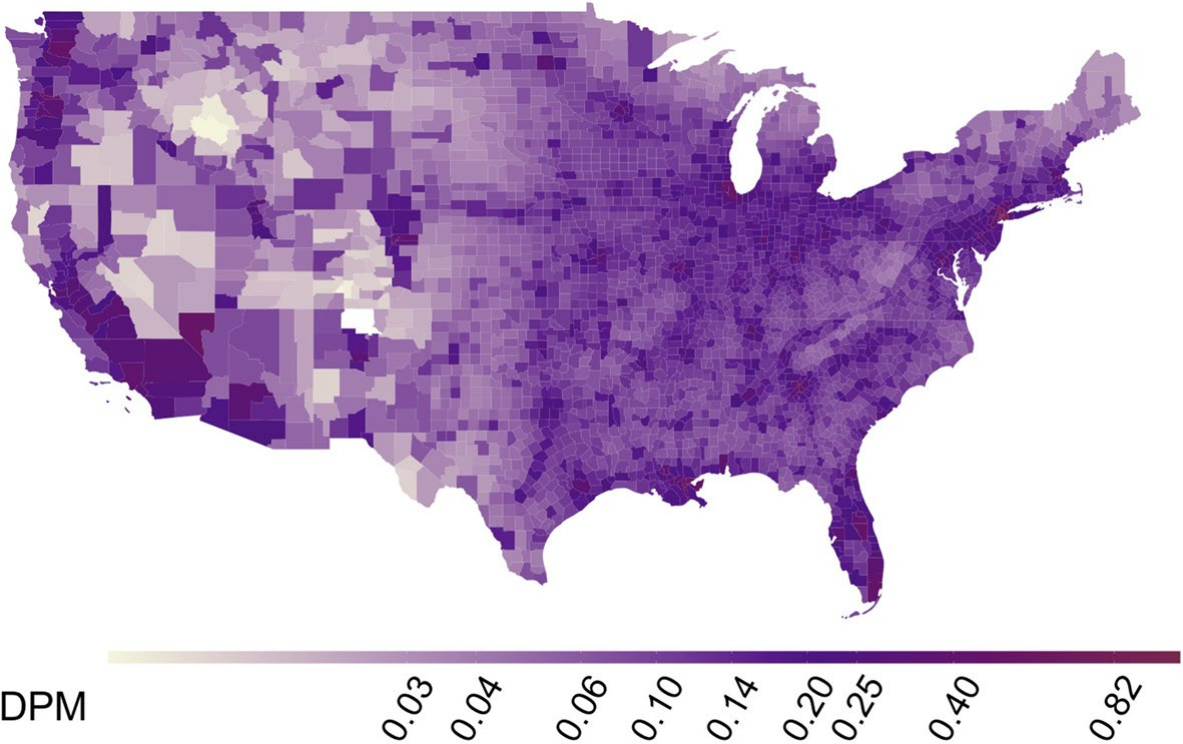
Spatial distribution of DPM concentration across contiguous U.S. counties (μg/m^3^). The R Statistical Software version 4.0.5 was used to produce the map, using the package *lattice*

County-level annual average DPM concentration varied from 0.000202 to 1.72 μg/m^3^ with a nationwide median of 0.204 μg/m^3^. Elevated DPM concentration could be observed at specific points corresponding to cities (Fig. 1). Across New York metropolitan area counties, which was greatly impacted during the first wave of the pandemic, the average DPM concentration was 0.425 μg/m^3^.

**Fig. 2:**
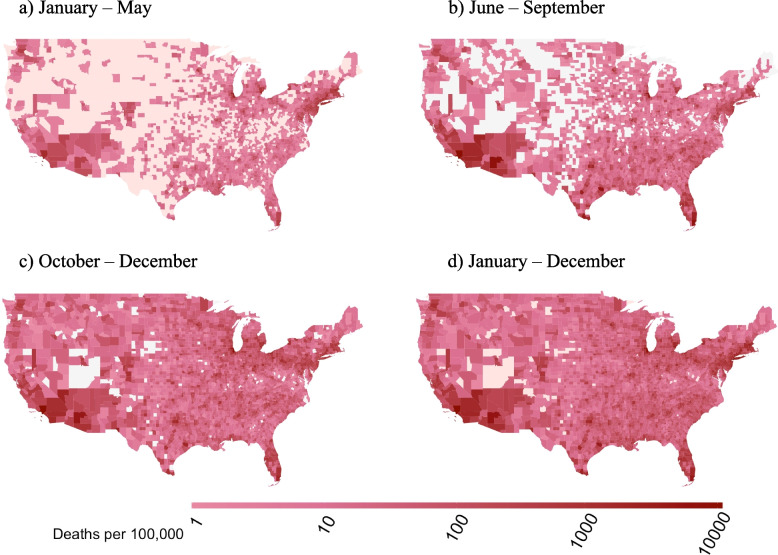
Spatial distribution of COVID-19 mortality rate for (**a**, top left) January-May, (**b**, top right) June-September, (**c**, bottom left) October-December, and (**d**, bottom right) all of 2020. The R Statistical Software version 4.0.5 was used to produce the maps, using the package *lattice*

During the January-to-May wave, the highest cumulative numbers of COVID-19 deaths were found in roughly the same regions as elevated DPM (Fig. 2a). As 2020 progressed, most counties experienced a higher mortality rate. The New York region exhibited lower cumulative mortality rate during the October-to-December wave of our study (Fig. 2c), with a mean of 98 deaths per 100,000 compared with the January-to-May wave, which had a mean of 280 deaths per 100,000 (Fig. 2a). As shown in Fig. 2a and b, cumulative mortality rate increased substantially from the first wave to the second wave in the Southeast region. In the West region, New Mexico, Arizona and California displayed the same pattern as the Southeast, with a large increase during the second wave. For the September-to-December wave, COVID-19 mortality rate increased across almost all of the US, exhibiting nearly the same pattern as for the all-year distribution (Fig. 2c and d).

At a global level, all models demonstrated a statistically significant association between long-term average DPM concentration and COVID-19 mortality rate for the first 9 months of 2020, as represented by the January-to-May and June-to-September waves (Table 3). SLM and SEM produced slightly higher associations for the June-to-September wave. For the October-to-December wave, none of the global models were found to produce positive associations or to be statistically significant. For the entire year, both the OLS and SLM produced positive associations, while the SEM produced a negative association.

**Table 3:** Mortality rate per 100,000 people per change in independent variable

Variable	January – May, 2020				June – September, 2020				October– December, 2020				January– December, 2020			
	OLS	SLM	SEM	GWR	OLS	SLM	SEM	GWR	OLS	SLM	SEM	GWR	OLS	SLM	SEM	GWR
DPM^a^	2.920 (1.237, 4.603)	1.710 (0.08608, 3.334)	1.845 (−0.2474, 3.9364)	3.125 (−23.36 – 76.94)	3.478 (1.561, 5.395)	3.275 (1.399, 5.152)	3.689 (1.416, 5.963)	1.109 (−29.99 – 72.56)	3.314 (−1.292, 7.920)	1.740 (−2.377, 5.858)	0.3395 (−5.468, 6.147)	−5.329 (− 160.6 – 53.56)	8.896 (3.841, 13.95)	4.293 (−0.2126, 8.798)	0.8571 (− 5.602, 7.316)	−15.65 (− 136.7 – 72.87)
Black^b^	52.14 (43.96, 60.32)	37.77 (29.66, 45.88)	43.18 (32.43, 53.94)	38.11 (− 956.2 – 595.0)	108.1 (98.87, 117.3)	85.29 (75.18, 95.40)	103.9 (92.81, 115.0)	−54.83 (− 1278 – 358.3)	−31.38 (− 50.25, − 12.51)	−16.79 (− 33.72, 0.1476)	−32.91 (− 58.83, − 6.991)	−36.54 (− 4541 – 12,079)	123.9 (100.3, 147.5)	67.42 (45.86, 88.98)	104.4 (73.23, 135.6)	88.63 (− 3762 – 1420)
Hispanic^c^	–	–	–	–	65.02 (54.36, 75.68)	51.15 (40.43, 61.86)	60.29 (47.53, 73.05)	68.11 (− 240.0 – 325.0)	–	–	–	–	101.1 (78.09, 124.1)	66.13 (45.32, 86.94)	105.5 (74.06, 136.9)	68.79 (− 1217 – 704.7)
American Indian^d^	30.04 (13.28, 46.80)	26.98 (10.85, 43.11)	25.95 (7.221, 44.69)	13.27 (− 3722 – 639.9)	–	–	–	–	137.1 (97.25, 176.9)	84.24 (48.51, 120.0)	71.98 (28.02, 115.9)	−72.70 (− 1973 – 3011)	216.5 (171.4, 261.6)	149.4 (109.0, 189.9)	146.9 (100.1, 193.6)	−71.81 (− 1544 – 7853)
Mining or Agriculture^e^	–	–	–	–	–	–	–	–	487.6 (402.1, 573.1)	243.4 (165.7, 321.0)	227.9 (130.6, 325.1)	33.60 (− 1995 – 2171)	410.8 (306.4, 515.2)	178.5 (84.88, 272.1)	115.1 (−3.324, 233.5)	112.7 (− 1838 – 2048)
Public Transport^f^	−505.3 (− 623.1, − 387.5)	− 720.9 (− 834.6, − 607.1)	− 840.4 (−964.8, − 715.9)	− 10.75 (− 2790 – 3850)	625.6 (484.9, 766.3)	735.5 (597.1, 873.8)	674.7 (525.9, 823.4)	− 132.9 (− 10,739 – 4070)	–	–	–	–	–	–	–	–
Drive to work^g^	–	–	–	–	−112.0 (− 211.5, − 12.45)	− 71.35 (− 168.8, 26.06)	−55.44 (− 156.7, 45.85)	35.78 (− 1487 – 1527)	–	–	–	–	–	–	–	–
Inactivity^h^	–	–	–	–	115.6 (92.02, 139.2)	99.16 (76.03, 122.3)	93.35 (68.16, 118.5)	43.88 (− 143.7 – 510.9)	–	–	–	–	204.4 (150.1, 258.6)	151.7 (102.9, 200.4)	191.5 (134.7, 248.3)	179.3 (− 267.5 – 1058)
Uninsured^i^	−48.26 (−69.45, − 27.07)	−42.74 (−63.14, − 22.35)	−35.58 (−64.24, −6.925)	16.25 (− 404.6 – 853.0)	–	–	–	–	–	–	–	–	–	–	–	–
Smoking^j^	−89.35 (− 123.4, − 55.30)	−80.49 (− 113.3, − 47.71)	−83.24 (− 126.6, − 39.85)	− 42.70 (− 1377 – 339.0)	–	–	–	–	174.4 (92.30, 256.5)	94.30 (20.54, 168.0)	175.4 (67.98, 282.9)	311.2 (− 1425 – 3479)	–	–	–	–
Obese^k^	–	–	–	–	–	–	–	–	56.68 (5.171, 108.2)	54.02 (7.962, 100.1)	63.99 (14.55, 113.4)	54.49 (− 550.6 – 899.5)	–	–	–	–
Age 65 or older^l^	–	–	–	–	–	–	–	–	88.62 (28.25, 149.0)	83.55 (29.57, 137.5)	90.38 (25.45, 155.3)	147.1 (− 602.9 – 909.2)	–	–	–	–
Income inequality^m^	3.888 (2.320, 5.456)	3.576 (2.067, 5.084)	2.855 (1.275, 4.434)	0.4881 (− 16.59 – 66.44)	–	–	–	–	–	–	–	–	11.64 (7.121, 16.09)	7.223 (3.246, 11.20)	9.199 (4.851, 13.55)	5.949 (−28.14 – 116.7)
Housing problems^n^	–	–	–	–	–	–	–	–	− 348.7 (− 414.4, − 283.0)	−188.3 (−247.6, − 128.9)	−189.1 (−261.4, −116.8)	−88.59 (− 2273 – 1276)	− 383.0 (−473.3, − 292.7)	−208.4 (− 289.6, −127.4)	− 233.1 (− 326.8, −139.4)	−142.9 (−256.0 – 1305)
Incomplete school^o^	–	–	–	–	1027 (739.5, 1315)	988.5 (706.9, 1270)	1051 (756.9, 1344)	471.6 (− 2395 – 7917)	–	–	–	–	–	–	–	–
Population density^p^	0.01311 (0.01215, 0.01407)	0.01328 (0.01234, 0.01422))	0.01574 (0.01480, 0.01667)	6.120 × 10^−4^ (−1.017 – 0.04822)	−0.01441 (− 0.01557, − 0.01325)	−0.01419 (− 0.01534, − 0.01305)	−0.01515 (− 0.01631, − 0.01398)	−1.948 × 10^− 3^ (− 0.05001 – 0.2498)	–	–	–	–	–	–	–	–
R^2^	0.37	0.42	0.45	0.65	0.41	0.43	0.44	0.59	0.16	0.33	0.31	0.49	0.15	0.32	0.32	0.44

Where cells are left blank, the forward stepwise variable selection process did not identify those variables for inclusion in the model. Ninety-five percent confidence intervals are provided for the OLS, SLM, and SEM models. Coefficients obtained from the GWR analysis are presented as median (min – max) across counties

Units: ^a^ deaths per 100,000 per IQR μg/m^3^ change in concentration, ^b^ deaths per 100,000 per fraction of the population that identifies as Black, ^c^ deaths per 100,000 per fraction of the population that identifies as Hispanic, ^d^ deaths per 100,000 per fraction of the population that identifies as American Indian, ^e^ deaths per 100,000 per fraction of the population that works in agriculture or mining, ^f^ deaths per 100,000 per fraction of the population that takes public transportation to work, ^g^ deaths per 100,000 per fraction of the population that drives to work, ^h^ deaths per 100,000 per fraction of the population that is inactive, ^i^ deaths per 100,000 per fraction of the population that is uninsured, ^j^ deaths per 100,000 per fraction of the population that smokes, ^k^ deaths per 100,000 per fraction of the population that is obese, ^l^ deaths per 100,000 per fraction of the population that is age 65 or older, ^m^ deaths per 100,000 per income inequality ratio, ^n^ deaths per 100,000 per fraction of the population that experiences housing problems, ^o^ deaths per 100,000 per fraction of the population that has not yet completed school, p deaths per 100,000 per change in population density people/mile^2^

OLS did not seem to be the most appropriate model to study spatial associations between COVID-19 mortality rate and DPM. Smaller associations for the spatial autoregression models compared with OLS suggested that the OLS covariates were positively biased due to spatial autocorrelation. Moran’s I and visual inspection of the residuals maps (Supplemental Fig. S1) indicated spatial clusters of high values and of low values. The Breusch-Pagan test provided support for heteroscedasticity of the residuals (p < < 10^− 6^) in the OLS models, which may have been partially attributed to spatial autocorrelation [28]. The SLM and SEM models provided modest improvements in model fit, as indicated by slightly higher values of coefficient of determination (R^2^). Model fit testing indicates that the SLM and SEM provided comparable fits, based on the Lagrange multiplier test.

**Fig. 3:**
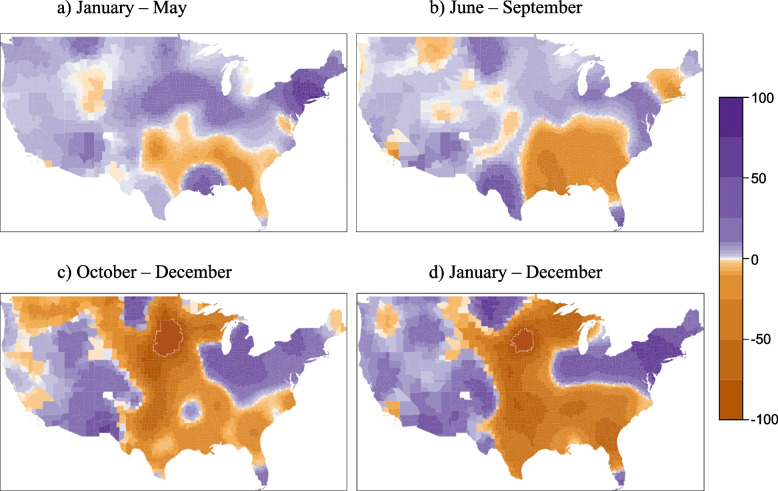
Map of associations between COVID-19 mortality rate and long-term DPM concentration for U.S. counties. The R Statistical Software version 4.0.5 was used to produce the map, using the package *lattice*

**Fig. 4:**
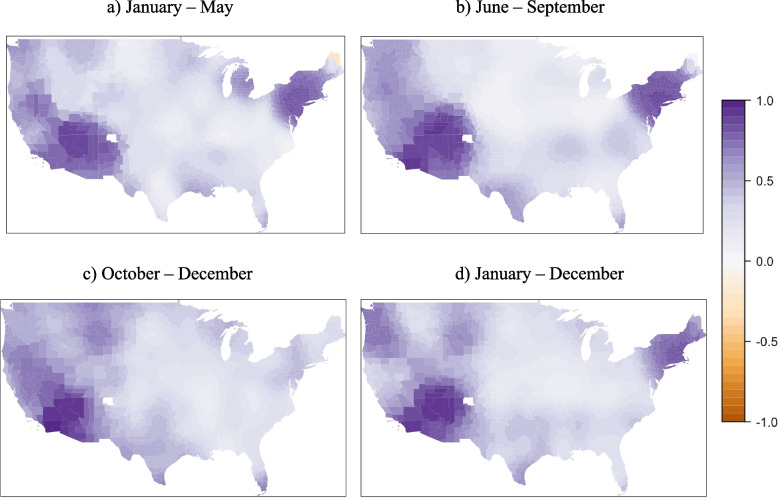
Spatial distribution of local R^2^ for the GWR model for mortality rate. The R Statistical Software version 4.0.5 was used to produce the map, using the package *lattice*

The local spatial differences estimated using the GWR model are presented as a range of values (Table 3). The mean COVID-19 mortality rate – DPM association for the GWR is identical to that of the OLS, but overall R^2^ for the GWR indicates improved performance over all global models. Spatial distribution of the DPM coefficients indicates changing conditions across the country during the three parts of the year (Fig. 3). During the January-to-May wave, associations were mostly positive across the U.S. (Fig. 3a), up to an increase of 76.94 deaths per 100,000 for every interquartile range (IQR) increase in DPM concentration. During the June-to-September wave, about half of the contiguous US presented a positive association (Fig. 3b), while associations were more negative for the October-to-December wave (Fig. 3c). Year-round COVID-19 mortality rate associations with DPM were similar to those for the October-to-December wave, likely due to the large number of cases during that timeframe. Local variations in R^2^ across the waves showed high (> 70%) values in the Northeast and Southwest during the January-to-May and June-to-September waves and in the year-long model. High R^2^ persisted into the October-to-December wave for the Southwest, albeit with a smaller area (Fig. 4). Low values of R^2^ (< 40%) were observed in the areas with greatest decrease in mortality with increasing DPM concentration, suggesting much greater uncertainty in those associations than in the positive ones seen in the New York area during the first wave. Moreover, COVID-19 mortality rate was statistically significantly associated with DPM concentration during the January-to-May and June-to-September waves but not during the October-to-December wave.

**Fig. 5:**
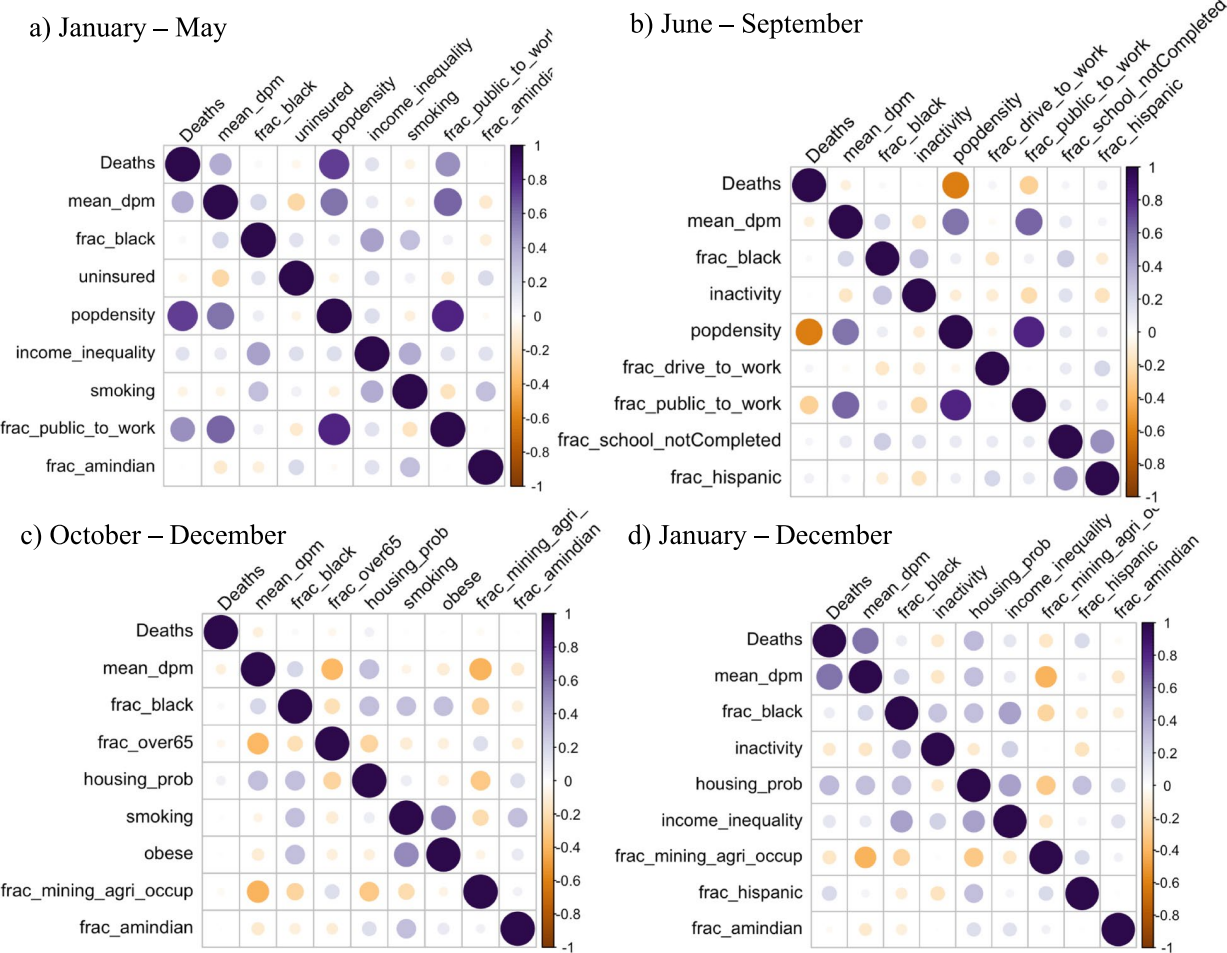
Pearson correlation matrix for mortality rate for (**a**, top left) January-May, (**b**, top right) June-September, (**c**, bottom left) October-December, and (**d**, bottom right) all of 2020

Among all potentially confounding covariates incorporated in the global models, fraction Black race and fraction American Indian ethnicity were statistically significantly positive in all global models. In addition to these two covariates, inactivity was significant in the June-to-September and October-to-December waves and in the year-long model, and the confounders Hispanic, Mining or Agriculture, Public Transportation, Time to Work, Income Inequality, and Population Density were significant at different time periods of the model. A negative relationship was found for smoking for the January-to-May wave, while a strong positive association was obtained for the October-to-December wave. In evaluations of the effects of smoking on COVID-19 incidence or mortality, inconsistent results have been found in the literature [1, 27, 31]. Benowitz et al. [1] highlighted the need for further investigations to better understand the mechanisms and effect of smoking on COVID-19 related outcomes. Correlation analysis suggests that DPM was moderately correlated with COVID-19 mortality rate in the January-to-May wave (Fig. 5a) and for the year-long (Fig. 5d) model, but that correlation was reduced in the June-to-September (Fig. 5b) and October-to-December (Fig. 5c) waves. Population density was correlated with COVID-19 mortality rate (ρ = 0.74) and DPM (ρ = 0.59) in the January-to-May wave. Fraction using public transportation was moderately correlated with COVID-19 mortality rate (ρ = 0.48) and DPM (ρ = 0.62) in the January-to-May wave. Therefore, population density and public transportation usage had the potential to act as confounders in a model testing the association between COVID-19 mortality and DPM concentration for the January-to-May wave. A model including just DPM in the SLM and SEM produced effect estimates of 7.728 and 9.713 deaths due to COVID-19 per 100,000 people for an IQR change in DPM (with R^2^ = 0.2 for both models), respectively. Inclusion of the covariates in the model produced effect estimates of 1.710 and 1.845 deaths due to COVID-19 per 100,000 people for an IQR change in DPM (with R^2^ = 0.42 and 0.45), respectively. These differences suggest that the final models controlled for those confounders. In the model that only included the covariates, the effect estimates for population density and use of public transportation were slightly lower than in the full model, while R^2^ for the SLM and SEM were the same as for the models including DPM.

Detailed results from the models examining associations between CFR and DPM are included in the Supplemental Material (see Supplemental text, Supplemental Table S1, and Supplemental Figs. S2-S5). The relationships between CFR and DPM were similar to associations between mortality rate and DPM at each wave and throughout 2020. During the January-to-May wave, associations were positive and strongest in the Northeast. Associations were visible through parts of the Midwest and the Pacific Northwest. Associations persisted in the Northeast for the June-to-September and October-to-December waves, but the magnitude of the associations was lower than for January-to-May. Negative associations were observed across the Southern and Mountain states for the June-to-September and October-to-December waves. Model fit (R^2^) was consistently lower for the CFR models across model type and wave compared with the models testing associations between mortality rate and DPM.

## Discussion

Our study analyzed the spatial correlation of COVID-19 mortality rate and case fatality rate with long-term DPM concentration as a surrogate for exposure across the continental United States during three waves of the COVID-19 pandemic during 2020. Our results suggested that long-term exposure to DPM may have been an important factor in COVID-19 mortality during the first two waves of the disease and that long-term DPM exposure may have been more highly influential during the January-May wave. Sidell et al. [44] examined associations between air pollution exposure and COVID-19 incidence for monthly and annual averages of PM_2.5_, nitrogen dioxide (NO_2_), and ozone (O_3_) over four waves corresponding to those in our study plus January-February, 2021 for a Southern California cohort. They similarly observed that PM_2.5_ had a larger effect during the first wave and that the effect diminished over time. A spatial autocorrelation term was controlled for in these models, but Sidell et al. [44] did not incorporate local methods. Differences in the outcome variable and the specific exposure also necessitates a further examination of spatial and temporal patterns.

Our results indicate that the OLS model does not account for the spatial associations of COVID-19 mortality rate or CFR with DPM concentrations. These results are similar to those of Sidell et al. [44] and Mollalo et al. [30], although their studies considered COVID-19 incidence rate rather than mortality. Mollalo et al. [30] used OLS, SLM, SEM, and two versions of the GWR to model COVID-19 incidence and mortality for the time period of January 22-April 9, 2020 and found notable spatial associations of both COVID-19 incidence and mortality with several predictors. The study of Hendryx and Luo [18], covering the January-to-May wave, revealed strong associations of COVID-19 prevalence and mortality with long-term DPM and PM_2.5_ concentrations. Their study estimated a coefficient of 14.3-18.7 deaths per 100,000 U.S. residents for each increase of 1 μg/m^3^ in DPM concentration. Inflation of the DPM effect shown in their results is possibly due to the correlation between covariates and their mixed linear multiple regression model that does not account for spatial correlation. Stakhovych and Bijmolt [46] emphasized that correlated spatial errors lead to bias and uncertainty in the OLS results. Moreover, LeSage and Fischer [26] noted that spatial correlation in the OLS error terms is a sufficient motivation to employ spatial autoregression models for discerning spatial relationships between dependent and independent variables.

The spatial global models outperformed the OLS model fit for all models for both mortality metrics. This improved performance may be related to spatial autocorrelation. A difference in coefficients and R^2^ among the OLS, SLM, and SEM models was not observed for mortality rate during the June-to-September wave. Kim [21] reported an inflated effect of spatial autocorrelation on OLS predictor coefficients, suggesting less spatial autocorrelation during the June-to-September wave consistent with Bini et al. [2] and Smith and Lee [45].

Among the modeling techniques analyzed for our study, GWR provided the best model fit, based on estimated global R^2^. Our results revealed where and when local long-term exposure to DPM may have been associated with COVID-19 mortality, consistent with results from both Karaye and Horney [20] and Mollalo et al. [30] regarding patterns of local prevalence and local mortality of the disease based on local R^2^. Some areas in the Northeast and West regions presenting a high R^2^ in our mortality rate model align with Mollalo et al. [30] estimates for incidence rate. As noted by Fotheringham et al. [15], our GWR results illustrate the need to account for local phenomena.

Socio-economic disparity could explain the non-stationary effect of DPM exposure on COVID-19 mortality rate, due to drastic differences between contiguous areas. Socially vulnerable communities, including minoritized racial groups, have seen spatially associated COVID-19 incidence [20]. This is consistent with the strong association we observed for the fraction Black confounder in the mortality rate model (Table 3). Moreover, Paolella et al. [32] pointed out spatial associations among fine particulate matter concentration, health effects, and minoritized groups and found out that finer spatial resolution revealed substantially higher fine particulate matter concentrations in Black and Hispanic communities.

The differences among associations of COVID-19 mortality rate and DPM concentrations found by the SLM and SEM for the year-long time period, when SLM was demonstrated to be more significant by a Lagrange test, helped to illustrate that neighboring effects were more relevant in modeling the spatial relationship with COVID-19 deaths than unobserved latent variables contained in the error term. Counties near other counties with high COVID-19 incidence are likely to have higher incidence. Nonetheless, since the weighting matrix chosen for our study was based on spatial adjacency, the county size differences between the Eastern and Western U.S. may have affected the parameter estimates creating more uncertainty in the larger counties [6]. Some variability in the association between COVID-19 and DPM exposure within counties might not have been captured, although DPM sources are more likely to be found in urban areas. However, since the SLM and SEM for the year-long time period were not statistically significant, other models should be considered when data are combined across multiple waves.

Several limitations of this study need to be acknowledged with respect to the input data. It is possible that, with more data and/or more time, the associations would disappear. Exposure measurement error could bias the results [52]. Our spatial modeling approach is intended to account for spatial exposure measurement errors. However, errors from applying cross-sectional analyses persist. Although we studied different waves of the disease, our models were not truly longitudinal. Long-term exposure to DPM was estimated using concentrations from 2018. The dataset likely includes higher DPM concentrations than for 2020 given reduced driving patterns during 2020 and, to a lesser extent, fleet turnover. This suggests that the magnitude of the effects of DPM calculated by our study and these other studies were underestimated. Widely reported undercounting of cases and deaths during the January-to-May wave would further contribute to this underestimation [13].

The set of potential confounders employed in our models was chosen to evaluate the influence of factors other than DPM potentially associated with COVID-19 outcomes [54]. However, it was impossible to represent all influential factors in the relationship between each wave of COVID-19 mortality and long-term DPM concentrations, so uncertainty in the potential for confounding existed [18, 54]. Furthermore, the study was designed at county level. Spatial variation within counties was not captured, and the difference in county size could have caused uncertainty since the weighting matrix defined for our SLM, SEM and GWR accounted for spatial adjacency. Therefore, associations at scales finer than county-level, including individual- and neighborhood-level associations, could not be inferred [54]. Despite these limitations, our study included a rigorous analysis of spatial relationships for different time periods and tested a variety of potential confounders to minimize these limitations.

## Conclusions

Our study built on previous findings by exploring associations of COVID-19 mortality rate with long-term DPM concentrations across the first three waves of the pandemic. In doing so, our models provided a picture in which long-term DPM exposure may have influenced COVID-19 mortality during the early stages of the disease, as observed specifically for the periods of January-to-May and June-to-September, 2020. Waning influence of DPM during the October-to-December wave suggested that person-to-person disease transmission regardless of past DPM exposures may have become more influential in the spread of COVID-19 and in mortality rates once the Coronavirus became widespread throughout the U.S. Further investigation might focus on factors associated with COVID-19 mortality rate during the October-to-December wave. Although COVID-19 data were available beyond this period, the introduction of vaccines during 2021 were likely to have been so influential that combination of the 2 years of data may have produced misleading conclusions.

## Supplementary Information

**Additional file 1: Table S1.** Deaths per 100 confirmed cases per change in independent variable. **Figure S1.** Spatial distribution of residuals for OLS models for mortality rate. **Figure S2.** Spatial distribution of COVID-19 case fatality rate for (a, top left) January-May, (b, top right) June-September, (c, bottom left) October-December, and (d, bottom right) all of 2020. **Figure S3.** Map of associations between COVID-19 case fatality rate and long-term DPM concentration for U.S. counties. **Figure S4.** Spatial distribution of local R^2^ for the GWR model for case fatality rate. **Figure S5.** Pearson correlation matrix for case fatality rate for (a, top left) January-May, (b, top right) June-September, (c, bottom left) October-December, and (d, bottom right) all of 2020.

## Data Availability

The datasets supporting the conclusions of this article are in the following repositories: COVID-19 mortality data can be found in the Johns Hopkins University Global Coronavirus (COVID-19) Database, https://data.world/covid-19-data-resource-hub/covid-19-case-counts/workspace/file?filename=COVID-19+Cases.csv. Diesel particulate matter data can be found in the U.S. Environmental Protection Agency 2014 National Air Toxics Assessment, https://www.epa.gov/national-air-toxics-assessment/2014-nata-assessment-results#self. Community characteristics data can be found in the U.S. Census Bureau 2014-2018 American Community Survey, https://data.census.gov/cedsci/table?d=ACS%205-Year%20Estimates%20Data%20Profiles&g=0400000US22.050000&tid=ACSDP5Y2018.DP05&hidePreview=false&tp=true&moe=true&vintage=2018 Community characteristics data can also be found in the [42] County Health Rankings, http://www.countyhealthrankings.org.

## Abbreviations

ACS: American Community Survey
AERMOD: American Meteorological Society/Environmental Protection Agency Regulatory Model
AIC: Akaike information criterion
CHR: County Health Rankings
COVID-19: Coronavirus disease 2019
DPM: Diesel particulate matter
EPA: Environmental Protection Agency
GWR: Geographically weighted regression
ICU: Intensive care unit
NATA: National Air Toxics Assessment
NO_2_: Nitrogen dioxide
OLS: Ordinary least squares
O_3_: Ozone
PM_2.5_: Particulate matter with a nominal diameter of 2.5 μm
R^2^: Coefficient of determination
SEM: Spatial error model
SLM: Spatial lag model
U.S.: United States
