# Supplementary material for: Spatial associations of long-term exposure to diesel particulate matter with seasonal and annual mortality due to COVID-19 in the contiguous United States
Source: BMC Public Health. 2023 Mar 3;23:423. doi: 10.1186/s12889-023-15064-5 (PMC9982169; doi:10.1186/s12889-023-15064-5)
Supplement: Supplementary file 1 — Additional file 1: Table S1. Deaths per 100 confirmed cases per change in independent variable. Figure S1. Spatial distribution of residuals for OLS models for mortality rate. Figure S2. Spatial distribution of COVID-19 case fatality rate for (a, top left) January-May, (b, top right) June-September, (c, bottom left) October-December, and (d, bottom right) all of 2020. Figure S3. Map of associations between COVID-19 case fatality rate and long-term DPM concentration for U.S. counties. Figure S4. Spatial distribution of local R2 for the GWR model for case fatality rate. Figure S5. Pearson correlation matrix for case fatality rate for (a, top left) January-May, (b, top right) June-September, (c, bottom left) October-December, and (d, bottom right) all of 2020. [file 12889_2023_15064_MOESM1_ESM.docx]

**Supplemental Information: Spatial Associations of Long-term Exposure to Diesel Particulate Matter with Seasonal and Annual Mortality Due to COVID-19 in the Contiguous United States**

Martine Elisabeth Mathieu^1^, Joshua Gray^1,2^, Jennifer Richmond-Bryant^*1,2^

1 Center for Geospatial Analytics, North Carolina State University, Raleigh, NC 27695-8008

2 Department of Forestry and Environmental Resources, North Carolina State University, Raleigh, NC 27695-8008

*Case Fatality Rate*

During the January-May wave, the highest cumulative numbers of COVID-19 deaths were found in roughly the same regions as elevated DPM (Supplemental Figure S2). As 2020 progressed, most counties experienced a higher mortality rate. The New York region exhibited elevated CFR during each wave, with 6.04% of cases resulting in death for January-to-May, 5.95% for June-to-September, and 7.09% for October-December. Average CFR across the U.S. during each wave, was 3.33% for January-to-May, 2.09% for June-to-September, and 2.18% for October-December (Supplemental Figure S2a).

At a global level, the OLS, SLM, and SEM for the January-to-May, June-to-September, and October-to-December waves demonstrated a statistically significant association between long-term average DPM concentration and COVID-19 CFR, but the year-long models did not (Supplemental Table S1). Associations for the OLS, SLM, and SEM were three-five times higher for the January-to-May wave compared with the subsequent waves. For the entire year, none of the three global models produced statistically significant associations between COVID-19 CFR and DPM.

The local spatial differences estimated using the GWR model are presented as a range of values (Supplemental Table S1). For each wave and the entire year, R^2^ for GWR indicates improved performance over all global models. At the same time, R^2^ for the CFR-DPM model was consistently lower than R^2^ for the mortality rate. Spatial distribution of the DPM coefficients indicates changing conditions across the country during the three waves (Supplemental Figure S3). During the January-May wave, associations were mostly positive across the U.S. (Supplemental Figure S3a), up to an increase of 2.66 deaths per 100 cases for every IQR increase in DPM concentration. For the June-to-September and October-to-December waves, about half of the contiguous US presented a positive association with a maximum increase of 2.69 and 2.40 deaths per 100 cases for every IQR increase in DPM concentration (Supplemental Figure S3b). Positive associations were observed in the Northeast throughout the year. Associations were lower for the October-to-December wave compared with the June-to-September wave (Supplemental Figure S3c). Negative associations were observed in the Southeast, Southwest, and Mountain states. Year-round associations for CFR were similar to those for the October-December wave, likely due to the large number of cases during that timeframe.

During the January-to-May wave, R^2^ was low throughout the country (Supplemental Table S1 and Supplemental Figure S4a). Local variations in R^2^ across the waves showed somewhat higher values in the Southwest and Mountain states during the June-to-September and October-to-December waves and in the year-long model (Supplemental Figures S4b-d). Lower R^2^ for the CFR-DPM models compared with the mortality rate-DPM models suggested much greater uncertainty in the CFR models, potentially due to greater uncertainties built into the CFR metric.

Among all potentially confounding covariates incorporated in the models, unemployment and income inequality were statistically significantly positive in all global models. Public transportation was statistically significantly positive in the global models for the June-to-September and October-to-December waves and in the year-long model. None of the covariates was highly correlated with both DPM and CFR (Supplemental Figure S5), so confounding was not suspected.

Table S1. Deaths per 100 confirmed cases per change in independent variable. Where cells are left blank, the forward stepwise variable selection process did not identify those variables for inclusion in the model. Ninety-five percent confidence intervals are provided for the OLS, SLM, and SEM models. Coefficients obtained from the GWR analysis are presented as median (min – max) across counties.

| **Variable** | **January – May, 2020** | | | | **June – September, 2020** | | | | **October– December, 2020** | | | | **January– December, 2020** | | | |
| --- | --- | --- | --- | --- | --- | --- | --- | --- | --- | --- | --- | --- | --- | --- | --- | --- |
|  | OLS | SLM | SEM | GWR | OLS | SLM | SEM | GWR | OLS | SLM | SEM | GWR | OLS | SLM | SEM | GWR |
| DPM^a^ | 0.8909  (0.5540,  1.228) | 0.7422  (0.4082,  1.076) | 0.8856  (0.5079,  1.263) | 0.9705  (-1.247 – 2.657) | 0.2991  (0.1683,  0.4299) | 0.1579  (0.03871,  0.2771) | 0.1923  (0.02905,  0.3555) | 0.2335  (-0.8174 – 2.694) | 0.2713  (0.1286,  0.4140) | 0.1417  (0.009753,  0.2736) | 0.1587  (-0.01694,  0.3343) | 0.1745  (-1.487 – 2.400) | 0.009163 (-0.2752, 0.2935) | -0.01557  (-0.0731, 0.04197) | -0.00491 (-0.0857, 0.0759) | -0.1748  (-1.654 – 0.9804) |
| Hispanic^b^ | - | - | - | - | 0.8058  (0.2819,  1.330) | 0.3879  (-0.0874,  0.8632) | 0.09847  (-0.6446,  0.8415) | -0.1888  (-10.82 – 7.578) | - | - | - | - | - | - | - | - |
| Pacific Islander^c^ | -207.6  (-323.0,  -92.23) | -179.2  (-292.9,  -65.52) | -179.4  (-297.7,  -61.13) | -146.2  (-939.1 – 231.7) | -109.5  (-154.0,  -65.03) | -56.69  (-97.05,  -16.33) | -34.93  (-78.58,  8.719) | -42.52  (-977.1 – 488.3) | -120.4  (-168.3,  -72.48) | -68.19  (-112.4,  -23.97) | -44.34  (-92.24,  3.562) | -44.83  (-1058 – 496.1) | -75.81  (-100.25, -51.36) | -48.64  (-71.07,  -26.21) | -39.34  (-63.19,  -15.50) | -48.35  (-628.9 – 276.8) |
| Mining or agriculture^d^ | -15.63  (-22.46,  -8.801) | -13.10  (-19.85,  -6.347) | -14.97  (-22.38,  -7..555) | -15.65  (-69.39 – 21.99) | -8.389  (-10.98,  -5.796) | -5.190  (-7.558,  -2.822) | -6.821  (-9.777,  -3.865) | -3.487  (-59.35 – 29.05) | -5.024  (-7.841,  -2.207) | -3.050  (-5.655,  -0.4452) | -4.183  (-7.419,  -0.9470) | -0.8727  (-102.0 – 102.8) | - | - | - | - |
| Construction^e^ | -4.294  (-22.49,  13.91) | -3.803  (-21.73,  14.12) | -4.015  (-22.70,  14.67) | 4.647  (-180.8 – 71.01) | - | - | - | - | - | - | - | - | - | - | - | - |
| Public transport^f^ | - | - | - | - | 5.613  (0.6366,  10.59) | 1.794  (-2.720,  6.308) | 0.5321  (-5.824,  6.888) | -1.797  (-158.7 – 38.20) | 19.05  (13.65,  24.45) | 10.83  (5.822,  15.84) | 11.09  (4.287,  17.89) | -1.706  (-119.9 – 108.8) | 12.03 (9.261, 14.80) | 8.067  (5.513,  10.62) | 7.915 (4.455, 11.38) | 5.091  (-133.6 – 80.08) |
| Commuting time^g^ | - | - | - | - | 24.15  (14.67,  33.63) | 11.87  (3.238,  20.05) | 11.81  (1.397,  22.22) | 11.29  (-86.30 – 80.21) | 23.45  (13.15,  33.75) | 12.07  (2.540,  21.60) | 12.27  (0.9157,  23.62) | 11.18  (-218.2 – 90.53) | - | - | - | - |
| Inactivity^h^ | - | - | - | - | - | - | - | - | - | - | - | - | 1.722 (0.8800, 2.564) | 1.414  (0.7028,  2.126) | 1.965 (1.157, 2.773) | 2.132  (-17.75 – 15.11) |
| Uninsured^i^ | - | - | - | - | - | - | - | - | 2.964  (1.339,  4.589) | 1.754  (0.2524,  3.256) | 2.072  (-0.1291,  4.273) | 1.182  (-30.45 – 30.16) | 3.052 (2.989, 3.114) | 1.802  (1.022, 2.581) | 2.441 (1.312, 3.570) | 2.009  (-16.61 – 15.33) |
| Income inequality^j^ | 0.00672  (-0.2892,  0.3027) | 0.01136  (-0.2801,  0.3028) | 0.01029  (-0.2970,  0.3176) | -0.1006  (-3.281 – 1.663) | 0.1831  (0.0824,  0.2838) | 0.1021  (0.01069,  0.1935) | 0.09505  (-0.0069,  0.197) | 0.04480  (-0.4832 – 1.035) | 0.1942  (0.08160,  0.3068) | 0.1208  (0.01690,  0.2247) | 0.1115  (-0.00212,  0.2251) | 0.08418  (-1.932 – 1.634) | 0.1175  (-3.048, 3.283) | 0.07689  (0.02176,  0.1320) | 0.1152 (0.05570, 0.1746) | 0.05728  (-1.155 – 1.172) |
| Housing problems^k^ | - | - | - | - | - | - | - | - | - | - | - | - | -2.568  (-3.342,  -1.795) | -2.178  (-3.251,  -1.105) | -3.256  (-4.488,  -2.024) | -1.518  (-29.04 – 31.70) |
| Severe housing burden^l^ | 5.721  (-0.5588,  12.00) | 4.878  (-1.306,  11.06) | 4.095  (-2.583,  10.77) | -7.210  (-52.03 – 45.57) | - | - | - | - | - | - | - | - | - | - | - | - |
| Incomplete school^m^ | -53.15  (-90.14,  -16.16) | -49.38  (-85.82,  -12.94) | -50.70  (-89.31,  -12.09) | -65.06  (-358.6 – 93.21) | - | - | - | - | - | - | - | - | - | - | - | - |
| Unemployment^n^ | 26.47  (11.48, 41.46) | 20.48  (5.680,  35.28) | 22.92  (6.715,  39.13) | 32.68  (-25.87 – 88.77) | 18.95  (13.30,  24.60) | 11.97  (6.796,  17.14) | 16.81  (10.35,  23.27) | 18.10  (-15.01 – 135.3) | 19.02  (13.65,  24.45) | 11.94  (6.248,  17.63) | 14.46  (7.504,  21.42) | 17.11  (-41.23 – 164.5) | 11.28 (10.11, 12.45) | 7.713  (4.796,  10.63) | 9.315 (5.740, 12.89) | 8.475  (-42.62 – 76.90) |
| R^2^ | 0.050 | 0.079 | 0.076 | 0.13 | 0.10 | 0.26 | 0.25 | 0.32 | 0.088 | 0.22 | 0.21 | 0.33 | 0.093 | 0.24 | 0.24 | 0.42 |
| Units: ^a^ deaths per 100 confirmed cases per IQR μg/m^3^ change in concentration, ^b^ deaths per 100 confirmed cases per fraction of the population that identifies as Hispanic, ^c^ deaths per 100 confirmed cases per fraction of the population that identifies as Pacific Islander, ^d^ deaths per 100 confirmed cases per fraction of the population that works in agriculture or mining, ^e^ deaths per 100 confirmed cases per fraction of the population that works in construction, ^f^ deaths per 100 confirmed cases per fraction of the population that takes public transportation to work, ^g^ deaths per 100 confirmed cases per fraction of time spent commuting to work, ^h^ deaths per 100 confirmed cases per fraction of the population that is inactive, ^i^ deaths per 100 confirmed cases per fraction of the population that is uninsured, ^j^ deaths per 100 confirmed cases per income inequality ratio, ^k^ deaths per 100 confirmed cases per fraction of the population that experiences housing problems, ^l^ deaths per 100 confirmed cases per fraction of the population that pays more than 50% of income on housing, ^m^ deaths per 100 confirmed cases per fraction of the population that has not yet completed school, ^n^ deaths per 100 confirmed cases per fraction of the population that is unemployed. | | | | | | | | | | | | | | | | |


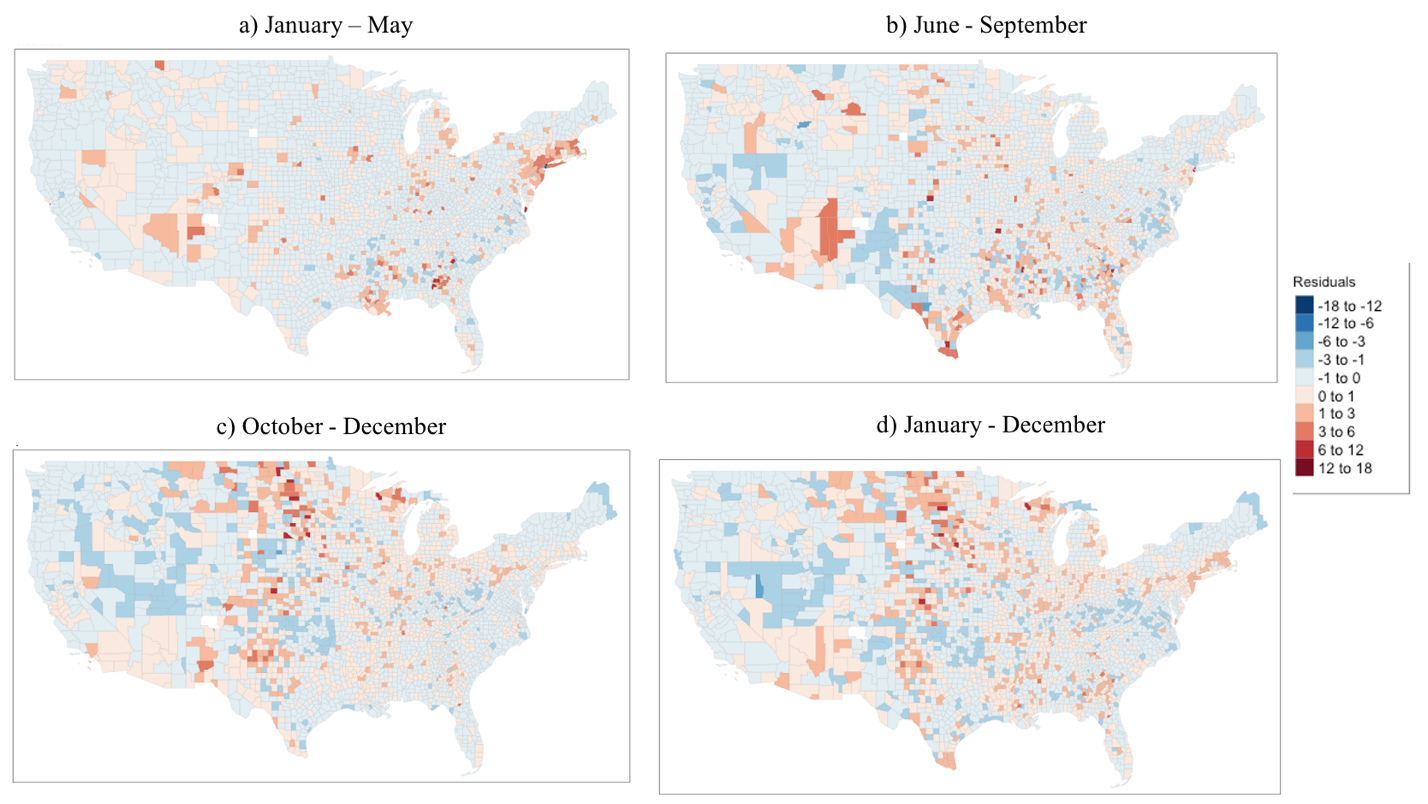


Figure S1. Spatial distribution of residuals for OLS models for mortality rate. The R Statistical Software version 4.0.5 was used to produce the map, using the package *lattice*.

Figure S2. Spatial distribution of COVID-19 case fatality rate for (a, top left) January-May, (b, top right) June-September, (c, bottom left) October-December, and (d, bottom right) all of 2020. The R Statistical Software version 4.0.5 was used to produce the map, using the package *lattice*.

Figure S3. Map of associations between COVID-19 case fatality rate and long-term DPM concentration for U.S. counties. The R Statistical Software version 4.0.5 was used to produce the map, using the package *lattice*.

Figure S4. Spatial distribution of local R^2^ for the GWR model for case fatality rate. The R Statistical Software version 4.0.5 was used to produce the map, using the package *lattice*.

Figure S5. Pearson correlation matrix for case fatality rate for (a, top left) January-May, (b, top right) June-September, (c, bottom left) October-December, and (d, bottom right) all of 2020.
